# Plant stress early detection through a low-cost multispectral device: Toward safer and more sustainable agricultural practices

**DOI:** 10.1016/j.isci.2026.116269

**Published:** 2026-06-14

**Authors:** Clemente Lauretti, Sara Cimini, Alessandro Zompanti, Christian Tamantini, Benedetta Pizziconi, Marzia Piemontese, Giorgio Pennazza, Marco Santonico, Laura De Gara, Loredana Zollo

**Affiliations:** 1 Unit of Advanced Robotics and Human-Centred Technologies, Departmental Faculty of Engineering, Campus Bio-medico University of Rome, Rome, RM, Italy; 2 Unit of Food Science and Nutrition, Departmental Faculty of Science and Bio-Technology, Campus Bio-Medico University of Rome, 00128 Rome, RM, Italy; 3 Unit of Electronics for Sensor Systems, Departmental Faculty of Engineering, Campus Bio-medico University of Rome, 00128 Rome, RM, Italy; 4 Institute of Cognitive Sciences and Technologies, National Research Council of Italy, 00196 Rome, RM, Italy; 5 Unit of Electronics for Sensor Systems, Departmental Faculty of Science and Bio-Technology, Campus Bio-Medico University of Rome, 00128 Rome, RM, Italy

**Keywords:** plant biology, plant physiology, plant pathology, agricultural instrumentation

## Abstract

While multispectral sensors offer a cost-effective and robust solution for monitoring plant responses to environmental stress, their limited spectral resolution, largely dependent on vegetation indices, can hinder accurate classification of stress severity using machine learning. This paper aims at overcoming these limitations by introducing a multispectral device for plant stress early detection that is 1) affordable for a wide range of end-users, 2) robust to environmental factors, 3) capable of automatically finding the most meaningful features that maximize the stress detection accuracy, and 4) capable of discriminating different plant stress severity. The device integrates a broadband LED and a VIS-NIR multispectral sensor to early predict plant stress through machine learning algorithms (i.e., SelectKBest, kNN, SVM, and LDA). It was trained on spectral measurements acquired from tobacco plants under salinity stress. The results demonstrated its high capability to discriminate with high accuracy different stress severity (average accuracy of 91.0 ± 3.1%).

## Highlights

• Commercial devices' limited spectral resolution hinders accurate stress classification
• The proposed device automatically selects features to maximize detection accuracy
• The 3D printed enclosure is robust to environmental factors for reliable measurements
• The proposed pipeline accurately discriminates different plant stress severity levels

## Graphical abstract

**Figure:**
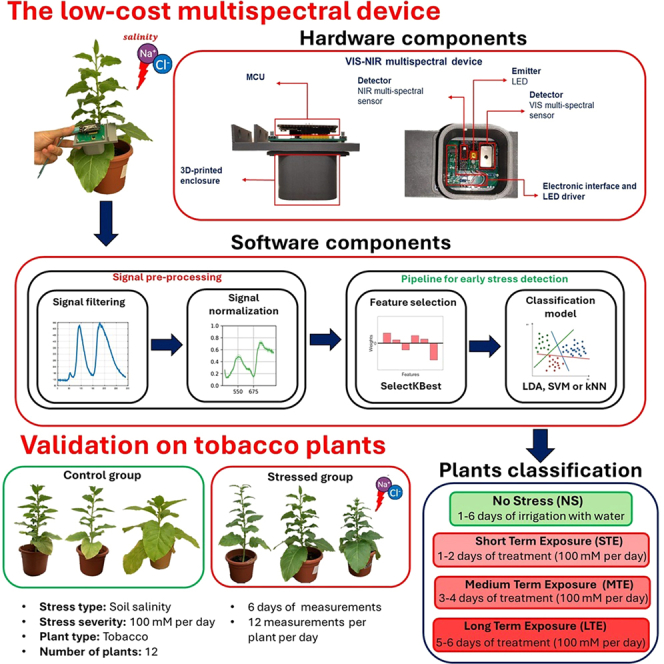

## Introduction

Plant diseases and stress are responsible for major economic losses in the agricultural industry worldwide. The Food and Agriculture Organization (FAO) highlights that biotic and abiotic stresses significantly impact crop yields, with substantial variations depending on factors such as the type of crop, geographic location, and the severity and duration of the stress conditions.^1^

Traditionally, growers rely on their experience, primarily through visual inspection, to determine when intervention is needed and to identify the most effective treatment.^2^ However, human judgment is inherently subjective and can be influenced by the individual’s experience and lighting conditions. Consequently, the farmer’s accuracy in promptly detecting stress conditions in plants may be compromised, leading to potential delays in intervention and suboptimal treatment decisions. Additionally, there is a clear difficulty for farmers in determining the physical, chemical, and biological changes in plants during the very early stages from the beginning of stress exposure. Saline stress is a significant challenge in agriculture, particularly in arid and semi-arid regions where natural conditions such as low rainfall and high evaporation rates lead to naturally high salt concentrations in the soil. Extensive irrigation practices induce soil salinization when soil drainage is lacking and the temperature is high. Coastal areas are also particularly vulnerable to salinity stress due to seawater intrusion into freshwater aquifers and direct salt deposits from ocean spray. When plants are exposed to saline conditions, they experience osmotic stress and ion toxicity, which disrupt their physiological processes. This stress leads to reduced water uptake, impaired photosynthesis, and stunted growth, ultimately resulting in decreased crop yields. Farmers in affected areas must adopt strategies such as selecting salt-tolerant crop varieties or implementing soil management practices to mitigate the impact. Failure to address saline stress can lead to substantial economic losses, as reduced crop productivity directly affects food supply and farmer livelihoods.

Given the importance of this decision-making process, there has been a pressing need in recent years to develop new technologies that are capable of early detecting stress conditions in plants and supporting farmer decisions for precise and targeted interventions.^3^

In the literature, advanced sensing technologies, primarily based on optical sensors, are generally used to support the growers’ decision by precisely evaluating the color, texture, stomata conductance, and other characteristics of the leaves that are directly correlated to the plant stress conditions.^4^

Wearable electrochemical sensors, such as patches and microneedles,^5^^,^^6^^,^^7^ on the other hand, provide a useful way to monitor plants with high accuracy and a fast response time. However, their invasiveness and specificity for a single physiological signal, such as glucose or electrical signals, limit their suitability for large-scale crop monitoring.

For proximal measurements, porometers and fluorometers are the most widely utilized diagnostic tools to non-invasively measure leaf stomata conductance, chlorophyll content, and photosynthetic activity.^8^^,^^9^^,^^10^ These parameters are directly correlated with the plant health status and can be used to early detect stress conditions in plants before a visual symptom is evident. An example of a commercially available porometer/fluorometer is the LI-COR Biosciences (Lincoln, NE, USA), followed by the SPAD meter (Minolta Co., Ltd., Osaka, Japan) and the MultispeQ,^11^ which use different peak emission wavelengths of LEDs to measure leaf chlorophyll content. Even though these devices represent the gold standard in evaluating plant health conditions, they are expensive tools that are not accessible to all growers. Moreover, the success of the measurement is impacted by the tool positions on the leaf, due to the small dimensions of their measuring area (2 mm × 3 mm for the SPAD meter and 7.5 mm × 7.5 mm for the LI-COR). Indeed, the distribution of chlorophyll across the leaves is not uniform and can be extremely different in definite patches due to localized disease or differences.

Other techniques for plant health status assessment are based on hyper- and multi-spectral sensing.^12^ Machine learning algorithms and computer vision techniques are then adopted to process this data, enabling the growers to make informed decisions about which crops necessitate target intervention.^13^^,^^14^^,^^15^

For remote sensing, hyper- and multi-spectral cameras are the most adopted, but they generally require consistent and uniform illumination across the scene to capture accurate and reliable spectral information.^16^^,^^17^ Variations in lighting conditions influenced by atmospheric factors, that are typical of outdoor environments such as agricultural ones, can impact the spectral distribution of the incoming light and introduce additional noise or distortions in the captured multispectral images^18^ and reduce the accuracy in detecting diseases and abiotic stresses.

Visible and near-infrared (VIS/NIR) spectroscopy is another promising solution for the evaluation of plant health relying on proximal measurements. VIS/NIR spectroscopy has been applied to studies of a large number of plants under different stress types (such as drought, salinity, nutrient deficiency, pathogen attack, and so forth) and correlated with plant chemical characteristics (such as chlorophyll content, nutrient levels, secondary metabolites, and so forth).^19^^,^^20^

Due to the high cost of high-resolution spectrometers and cameras, researchers have explored low-cost multispectral devices, capable of acquiring multiple wavelengths simultaneously, and tested them on a small range of plant types (Corn, Canola, and Soybean) and stress typology (drought and nitrogen deficiency).^21^^,^^22^^,^^23^^,^^24^

The majority of the low-cost multispectral devices proposed in the literature are based on cameras, whose measurement is directly impacted by external lighting conditions influenced by atmospheric factors.^13^^,^^25^ To cope with this issue, a few devices have been equipped with LEDs enclosed in dark boxes that come in close contact with the leaf, but they present a limited number of wavelengths (maximum 6 wavelengths^22^^,^^23^^,^^24^^,^^26^) and the resulting low spectral resolution may not be sufficient to discriminate with high accuracy different stress severities through machine learning approaches. Moreover, most of the devices adopt specific spectral indicators to evaluate plant health, regardless of the type of plant to be evaluated and the stress condition to be detected.^21^^,^^23^^,^^24^ These indices are mathematical combinations of surface reflectance at different wavelengths that highlight specific vegetation properties. For instance, the simple ratio (SR) and its modified version (MSR)^27^^,^^28^ measure ratios of reflectances that help estimate vegetation biomass or leaf area index. The normalized difference vegetation index (NDVI)^29^ and the enhanced vegetation index (EVI)^30^ are commonly used for monitoring plant health, biomass, and photosynthetic efficiency, with EVI providing additional benefits in high biomass regions by reducing atmospheric interference. Other indices such as the green NDVI (NDVIg)^31^^,^^32^ use green instead of red reflectance, offering insights into chlorophyll content, which is also considered an indirect parameter indicating plant vigor. Similarly, the structure insensitive pigment index (SIPI)^33^ uses NIR, Blue, and Red reflectance to determine the carotenoid to chlorophyll ratio, indicating plant senescence due to the chlorophyll degradation occurring during this process and the stresses in which chlorophyll and carotenoid levels decrease (references).

Even though all these indices could enable informed assessments of crop health, crucial for effective agricultural practices, the accuracy of plant health evaluation and the timeliness of stress detection may vary depending on the plant type and stress severity to be evaluated. An automatic method that finds the most meaningful features on a broad range of multispectral measurements (not limited to the spectral bands necessary for the computation of the aforementioned vegetation indices) may improve the accuracy and timelessness of the stress prediction, with a positive effect on agricultural practices and crop management.

Therefore, this paper aims to introduce a multispectral device ad-hoc designed for plant stress early detection, capable of overcoming limitations of current devices proposed in the literature, by providing: 1) an affordable solution for a wide range of end-users, 2) robustness to environmental factors such as illumination conditions 3) capability of automatically finding the most meaningful features depending on the plant type to be evaluated and stress condition and 4) capability to accurately discriminate between different plant stress severities, even at the early stage, before the visual symptom is evident to the growers.

The proposed multispectral device integrates a broadband light-emitting diode (LED) and a multispectral sensor (with 52 wavelengths) to gather information about the plant stress severity through predictive machine learning algorithms, i.e., SelectKBest automatic feature selection and kNN.

Moreover, the proposed device is more easily adaptable to different plant types compared to literature approaches since the relevant features of the multispectral data, which are most meaningful for stress detection, are not plant- or stress-specific as they are automatically extracted and selected through advanced algorithms (i.e., SelectKbest approach).

Finally, it is more accurate to discriminate plant stress at the very early stage, before the visual symptom is evident to the growers, since it has a better spectral resolution due to the integration of two different multi-spectral sensors opportunely placed into the case to cover a range of 340–1050 nm in the visible and near-infrared spectrum with a total of 52 wavelengths.

The proposed multispectral device has been experimentally validated on tobacco plants, which are among the most extensively used in laboratory settings as a model plant for understanding the mechanism of abiotic stress.^34^

The device has been trained to detect and classify salinity stress, even in its early stages.

The classification performance obtained through the proposed multispectral device has been compared to that achieved through the leaf porometer/fluorometer LI-COR. Moreover, the advanced algorithms for automatic feature selection were compared to the literature approach based on specific vegetation indices.

## Results and discussion

### Plant material

Tobacco (*Nicotiana tabacum*) seeds were sterilized by soaking in 70% ethanol for 2 min, followed by soaking in 5% bleach for 15 min. The seeds were then rinsed five times with sterilized water. Sterilized tobacco seeds were sown in soil at 25 ± 1°C with a photoperiod of 16-h light/8-h dark. The solution of sodium chloride (NaCl) was produced in double-distilled water (DDW) at concentrations of 100 mM and used for plant irrigation in order to induce salt stress. Measurements using the developed multi-spectral device and the LICOR system are performed two months after seed germination under both control and salt stress conditions.

### The proposed multi-spectral device for plant stress early detection

The developed multispectral device^35^ is engineered to withstand environmental conditions, ensuring reliable measurements that are not influenced by varying atmospheric conditions, unlike spectral cameras. It can be equipped with a handle for manual measurements or integrated into a robotic end-effector for automated plant stress early detection. Commercial components were used to minimize costs and simplify maintenance, while custom-designed printed circuit boards (PCBs) were implemented to optimize the assembly process and provide a cost-effective alternative to conventional commercial spectrometers used in agriculture for plant stress detection. The sensors, carefully selected and mounted on the device, capture a broader spectrum of wavelengths than other low-cost sensors used in the literature for similar applications. This enhanced wavelength range enables a more detailed analysis of the external characteristics of leaves and improves the assessment of stress conditions through machine learning algorithms. The following sections provide a comprehensive overview of the components, detailing both the hardware and software aspects that regulate sensor operations.

#### Hardware components

**Figure 1:**
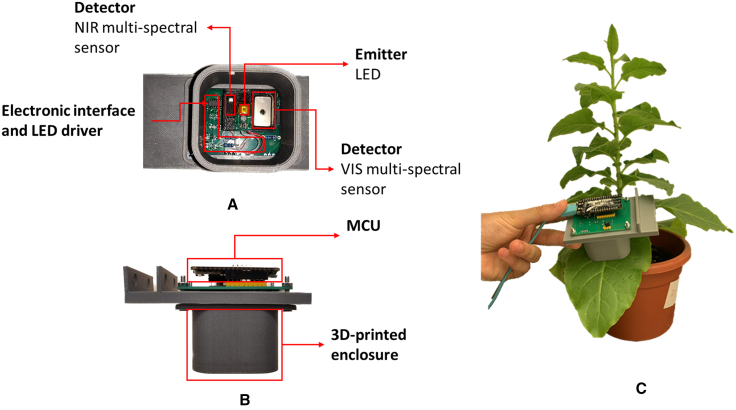
Hardware components of the multispectral device for plant stress detection^35^ (A) Bottom view of the overall assembly, (B) lateral view, and (C) example of usage.

The proposed device is composed of the following hardware components (see Figure 1).

• Broadband LED: The SMB1N-BB450 (Roithner Lasertechnik GmbH, Austria) is used as the light source in the device, illuminating the target leaf with a broad spectrum of light that spans wavelengths from 340 to 1050 nm. This spectrum is ideal for gathering spectral data essential for assessing the plant health status and detecting stress conditions in its early stages. The emission intensity of the LED is finely tuned to provide consistent lighting during data collection.
• Multispectral Sensors: The device includes two multispectral sensors, the C12880MA and the C14384MA (Hamamatsu Photonics K.K., Japan), which operate across the VIS-NIR spectra. These sensors are adept at acquiring the light reflected by leaves and utilize various spectral bands to harvest important spectral information indicative of different stress conditions in plants.
• Device Electronics: the electronics comprises microcontrollers, analog-to-digital converters, and additional circuitry necessary for data gathering, processing, and communication. The device operates on 5 V, consuming 235 mW in idle mode and 740 mW when active (i.e., during sensor data collection).
• 3D Printed Enclosure: A custom-made 3D-printed enclosure secures and supports all the hardware elements, providing a durable and compact framework. The interface between the enclosure and the leaves is specifically designed to allow the device to be placed gently on the leaves, avoiding damage. The enclosure not only shields the sensitive components from external elements but also ensures device stability and durability against lighting conditions during operation. It also includes a protective cover specifically engineered to protect the electronic parts and circuit board from moisture and other environmental contaminants.

The production of the device, encompassing the design, assembly, and testing phases for its primary hardware elements, took approximately one week. The total expenses for the materials, which include the electronic board, the multispectral sensors, and the 3D-printed casing, amounted to less than 600 euros.

#### Software components

**Figure 2:**
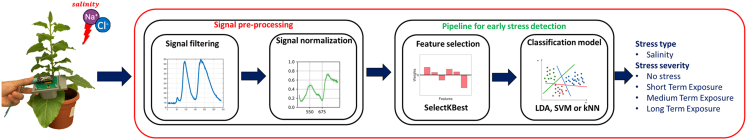
Pipeline for plant stress detection and classification through the proposed multispectral device The classification model could be based on the linear discriminant analysis (LDA), support vector machine (SVM) or k-nearest neighbors (kNN).

Besides the hardware elements, differently from,^35^ the device proposed in this work includes software components that are purposely designed to identify plant stress conditions using the collected multispectral data. These algorithms exploit distinct features and patterns in the spectral data. The process includes different steps, as depicted in Figure 2: i) signal pre-processing, ii) feature selection, and iii) plant stress classification.

##### Signal pre-processing

The first software component is dedicated to signal pre-processing. This includes enhancing the signal-to-noise ratio via signal filtering (employing a moving average filter with a 3-sample buffer) and normalizing the signal to mitigate any discrepancies or distortions arising from the light source. The normalized signal *R*_*n*_ is calculated as follows:

(Equation 1) $$R_{n}=\frac{R-D}{W-D}$$

where *R* represents the filtered leaf reflectance captured by the multispectral sensors under artificial illumination, *D* is the dark reference, i.e., the reflectance captured without artificial light, and *W* is the white reference, i.e., the reflectance from a white target under artificial light.^36^

##### Feature selection

The second software component is a feature selection step, namely the SelectKBest method, which selects *k* features based on their statistical significance according to the ANOVA F-value.^37^ This univariate feature selection method ranks features by their ability to differentiate between different stress severity classes, ensuring that only the most informative features are used for classification. By systematically varying the number of selected features, it is possible to assess the impact of dimensionality reduction on model performance and optimize the balance between retaining informative data and reducing noise or irrelevant information in the feature set.

##### Plant stress classification

The last software component is a supervised machine-learning model, which is intended to detect plant stress and classify its severity. To determine the optimal pipeline configuration, three different state-of-the-art machine-learning approaches have been compared in this work to identify the most suitable for plant stress detection and severity classification. They are: 1) the linear discriminant analysis (LDA), 2) the support vector machine (SVM), and 3) the k-nearest neighbors (kNNs). Each approach is detailed in Appendix.

### Plant stress classification pipeline through vegetation indices

As for the software pipeline shown in Figure 2, the process to classify stress conditions through vegetation indices includes the following steps, shown in Figure 2: 1) signal pre-processing, 2) feature selection, and 3) plant stress classification. However, unlike the pipeline shown in Figure 2, a feature extraction module is included before the feature selection to compute the vegetation indices widely recognized in the literature. The Appendix reports the vegetation indices adopted in this work to assess plant health status and detect stress conditions. These indices were carefully selected based on their ability to capture various physiological and biochemical traits related to plant stress responses, such as pigment content, water content, and other leaf characteristics. Through a combination of these indices, it is possible to capture a broad spectrum of plant physiological states, from healthy to stressed conditions.

### Plant stress classification pipeline through a porometer/fluorometer device

The pipeline adopted in this work to perform plant stress detection and classification through the porometer/fluorometer device (LICOR device) is similar to the one in Figure 2, but the first block is made up of a signal pre-processing and a feature extraction stage, both of which are automatically performed by the LICOR device.

The overall pipeline thus consists of the following steps: 1) signal pre-processing and feature extraction, 2) feature selection, and 3) plant stress classification.

The first step, i.e., signal pre-processing and feature extraction, is carried out directly by the LI-COR integrated processor, which derives three synthetic indices: stomatal conductance to water vapor (Gsw), the quantum yield of photosystem II (Φ_*PSII*_), and the electron transport rate (ETR). These parameters are closely linked to plant physiological processes, and their variations reflect the plant's interaction with environmental conditions. In particular, changes in Gsw indicate adjustments in stomatal regulation in response to factors such as water availability and salinity, while Φ_*PSII*_ and ETR provide insight into photosynthetic performance and its modulation under environmental stresses. The second and third steps, i.e., the feature selection and plant stress classification, are the same as those adopted for the classification pipeline shown in Figure 2. In the following, a description of the three retrieved indices, i.e., Gsw, Φ_*PSII*_, and TER, is provided.

Stomatal conductance to water vapor (Gsw) refers to the rate at which water vapor passes through the stomata of plant leaves. It is a key indicator of how open or closed the stomata are, which in turn affects both water loss and the exchange of gases (CO2 entering, water vapor, and O2 coming out) between the atmosphere and the plant's photosynthetic tissues. This parameter is crucial for understanding the plant water use efficiency, its thermoregulation ability through water evaporation and transpiration. It is defined as

(Equation 2) $$G_{sw}=\frac{E\cdot P_{\mathrm{atm}}}{VPD_{\mathrm{leaf-air}}}$$

where *E* is the transpiration rate (mmol *H*_2_*O m*^−2^ *s*^−1^), *P*_*atm*_ is the atmospheric pressure (kPa), and *VPD*_*leaf−air*_ is the vapor pressure deficit between the leaf and the air (kPa).

Quantum yield of photosystem II (Φ_*PSII*_) measures the efficiency of Photosystem II, one of the core components of the photosynthetic process. It represents the proportion of absorbed light energy that is used in photochemistry within Photosystem II. High values of Φ_*PSII*_ indicate that the plant is effectively using light energy for photosynthesis, whereas lower values suggest inefficiencies in the photosynthetic process, a situation usually induced by environmental stresses. It is defined as

(Equation 3) $$\Phi_{\mathrm{PSII}}=\frac{F_{m}^{\prime }-F_{s}}{F_{m}^{\prime }}$$

where $F_{m}^{\prime }$ is the maximum fluorescence measured in the light-adapted state and *F*_*s*_ is the steady-state fluorescence measured under light conditions.

Electron transport rate (ETR) is an estimate of the rate at which electrons are transported through the photosynthetic electron transport chain, which is crucial for the conversion of light energy into chemical energy during photosynthesis. ETR is used to assess the overall efficiency and capacity of the photosynthetic machinery. It helps in understanding how well a plant is performing photosynthesis, particularly under varying light conditions or environmental stresses. It is defined as

(Equation 4) $$ETR=\Phi_{\mathrm{PSII}}\times PPFD\times \alpha \times \beta$$

where Φ_*PSII*_ is the quantum yield of photosystem II, *PPFD* is the photosynthetic photon flux density (*μmol* photons *m*^−2^ *s*^−1^), *α* is the leaf absorbance factor (typically around 0.84), and *β* is the fraction of absorbed light that is distributed to photosystem II (often assumed to be 0.5).

### Experimental validation

The proposed device has been experimentally validated to detect and classify salinity stress in tobacco plants. In a controlled growth chamber setting, 12 tobacco plants were cultivated and divided into two distinct groups: 1) a control group (6 plants), which was irrigated solely with water, and 2) a treated group (6 plants), which received irrigation with a saline solution (100 mmol/L) for 6 days.

**Figure 3:**
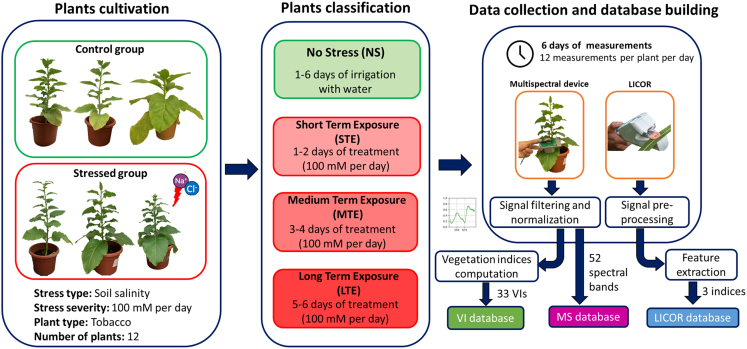
Experimental protocol for data acquisition, pre-processing and database building

Specifically, the validation process (shown in Figure 3) consisted of the following steps: 1) data acquisition, pre-processing, and building of the three databases, and 2) classification model training and testing.

#### Data acquisition, pre-processing, and database building

The first phase of the experimental validation consists of acquiring multispectral data and porometer/fluorometer signals from both the control and treated groups of plants.

Measurements were taken daily, with 12 measurements recorded per plant each day, allowing for a comprehensive analysis of the salinity impact over time. They were performed over short acquisition times, reducing the impact of potential LED heating. Under these conditions, no significant drift in the acquired signals was observed during the experimental campaign.

Multi-spectral and porometer/fluorometer scanning was made on four different leaves per plant, to provide a representative sampling of the plant while maintaining a reasonable acquisition time and ensuring experimental repeatability. Moreover, leaves were selected to ensure consistency across plants (e.g., similar developmental stage). For each leaf, measurements were performed in predefined areas, and multiple scans were acquired to reduce local variability and improve robustness. A total of three scans were measured at each position, and the measurements from the three positions were averaged as one sample.

Following data acquisition, the obtained multispectral signals underwent preprocessing steps as outlined in Figure 2. Instead, the porometer/fluorometer scannings were directly pre-processed by the LICOR-integrated processor to retrieve the three indices (gsw, Φ_*PSII*_ and ETR).

Subsequently, 3 databases were constructed: 1) one based on the features automatically selected from the 52 preprocessed multispectral bands, referred to as the MS database, 2) one based on the best vegetation indices computed from preprocessed multispectral signals, referred to as the VI database, and 3) one based on the indicators retrieved from the LICOR porometer/fluorometer, referred to as the LICOR database.

The features in the databases were labeled based on the severity of stress as shown in Figure 3: 1) features extracted from data acquired from the control group were labeled as the no stress (NS) class, 2) features extracted from data acquired from the treated group were labeled as Stressed; specifically, they were labeled as short-term exposure (STE) class, if calculated from data acquired during days 1–2 of treatment, they were labeled as medium-term exposure (MTE) class if calculated from data acquired during days 3–4 of treatment, and they were labeled as long-term exposure (LTE) class if calculated from data acquired during days 5–6 of treatment.

#### Classification model training and testing

A cross-validation strategy based on a leave-one-group-out (LOGO) scheme was adopted to evaluate the performance of different classification models and input datasets (i.e., LICOR, VI, and MS). In this framework, each group corresponds to a single plant, ensuring that all samples from one plant are excluded from the training set and used exclusively for testing. For each plant, 12 measurements per day were collected. This approach provides a more realistic estimate of the generalization capability of the models to unseen plants.

For each dataset, two classification tasks were considered: a binary task and a multiclass task (NS, STE, MTE, LTE). At each iteration of the LOGO procedure, the model was trained on data from all plants except one and tested on the held-out plant. This process was repeated for all plants, and the final performance was computed by aggregating predictions across all folds.

Feature selection was performed using a univariate ANOVA F-test. Feature ranking was computed once on the entire dataset before cross-validation, producing a global ordered list of features based on their discriminative power. An incremental feature selection strategy was then applied: Starting from the most informative feature, additional features were progressively included according to the ranking, without reordering previously selected ones. This allowed the systematic analysis of the impact of the number of features on model performance and to identify the optimal feature subset.

Three classification models were evaluated: LDA, SVM, and kNNs. For SVM and kNN, feature standardization was applied within a pipeline to ensure proper scaling of the input data. The SVM classifier employed a radial basis function kernel, while the kNN classifier was configured with *k*_*kNN*_ = 1.

The best-performing model was determined based on the highest average accuracy achieved on the cross-validation folds. This process was repeated for each of the input databases: LICOR, VI, and MS. Moreover, the classification pipeline was applied to two distinct classification tasks: binary classification and multiclass classification of stress levels. In the binary classification task, the goal was to distinguish between stressed and non-stressed plants. For the multiclass classification task, the objective was to classify plants into varying levels of stress, such as no stress (NS), STE, MTE, and LTE. Each pipeline was validated separately for both classification tasks, ensuring that the comparison of models and databases was consistent across both binary and multiclass contexts.

### Results of the data pre-processing and database building

**Figure 4:**
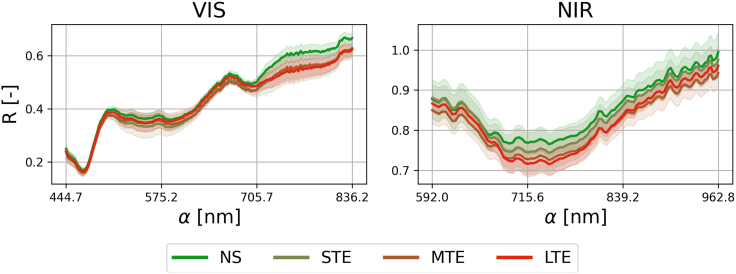
Raw data collected through the multispectral device at different stress levels: no stress (NS), short-term exposure (STE), medium-term exposure (MTE), and long-term exposure (LTE)

Figure 4 shows the raw multispectral signals, in the visible (VIS) and near-infrared (NIR) spectral regions, acquired from plants at different stress levels. In both spectra, there is a noticeable separation in reflectance between plants exposed to varying stress levels, particularly from 705 to 962 nm. Plants under LTE levels generally exhibit lower reflectance compared to those with no exposure to stress. This pattern suggests that stress affects pigment concentration and light absorption in this region. In the NIR spectrum, the differences between stress levels are even more pronounced than in the VIS range, with LTE to stress conditions showing decreased reflectance. From a physiological perspective, these variations are consistent with known plant responses to stress. Changes in the visible range are typically associated with variations in pigment concentration (e.g., chlorophyll degradation), while differences in the near-infrared region reflect structural and water-content alterations in the leaf.^19^^,^^38^ The marked sensitivity of the red-edge and NIR regions further supports their relevance for capturing early and progressive stress-related changes.

The overall trend indicates that both the VIS and NIR spectral regions provide valuable information for distinguishing between different stress levels in plants. However, it is worth noting that a slight discontinuity may be observed in the overlapping spectral region between the VIS and NIR sensors. This effect is mainly due to the intrinsic differences between the two sensors (e.g., sensitivity, spectral resolution, and optical characteristics), despite the normalization procedure based on white and dark references (Equation 1), which compensates for variations in sensor response and illumination conditions. It is important to highlight that the proposed classification pipeline does not rely on spectral continuity across adjacent wavelengths. Instead, it leverages the discriminative power of individual spectral features. In this context, the SelectKBest feature selection method identifies the most informative wavelengths, reducing the impact of potential inconsistencies in the overlapping region.

**Figure 5:**
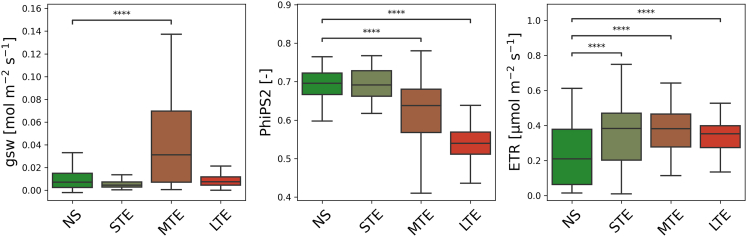
LICOR indicators collected at different stress levels: no stress (NS), short-term exposure (STE), medium-term exposure (MTE), and long-term exposure (LTE) Statistical significance was evaluated using the Mann-Whitney U test followed by Bonferroni correction. Asterisks denote statistical significance: ∗*p* ≤ 0.05, ∗∗*p* ≤ 0.01, ∗∗∗*p* ≤ 0.001, and ∗∗∗∗*p* ≤ 0.0001.

Figure 5 shows the variation of three indices derived from LICOR measurements, i.e., gsw, Φ_*PSII*_, and ETR, across the four applied levels of plant stress (NS, STE, MTE, and LTE). The Mann-Whitney U test with Bonferroni correction (*p*-value <0.01) was applied to assess statistical differences. Stomatal conductance (gsw) shows an irregular trend across stress conditions, with maximum values reached for medium-stressed plants. Φ_*PSII*_, which represents Photosystem II efficiency, shows a clear decreasing trend across stress conditions, indicating a decrease in the efficiency of the photosynthetic process. Electron transport rate (ETR) slightly increases as stress increases, providing statistically significant differences but a counterintuitive trend that contrasts with the overall pattern of physiological decline.

### Results of the feature selection and classification models

Table 1 reports the results obtained from the comparison among classification models in terms of 1) optimal number of features selected and 2) accuracy of classification for both binary and multiclass tasks, averaged on different classes to be detected. These models were evaluated across three collected databases: LICOR, VI, and MS databases.

**Table 1:** Results obtained from the comparison among classification models (LDA, SVM, and kNN) across different databases (MS, VI, and LICOR)

		Binary			Multiclass		
		LICOR	Vegetation indices	proposed MS	LICOR	Vegetation indices	proposed MS
# of Features	LDA	1	27	47	2	28	52
	SVM	1	30	43	3	20	48
	kNN	2	20	47	3	19	45
Accuracy [%]	LDA	82.2 ± 7.5	90.3 ± 4.4	92.3 ± 4.0	49.5 ± 6.7	67.3 ± 10.2	70.3 ± 11.2
	SVM	83.2 ± 7.4	88.7 ± 4.5	91.6 ± 5.2	56.5 ± 8.0	60.3 ± 6.8	70.1 ± 9.4
	kNN	82.0 ± 6.5	84.2 ± 3.8	89.1 ± 6.6	50.5 ± 5.5	48.3 ± 5.8	60.2 ± 10.3

In the binary classification task, which involves distinguishing between stressed and non-stressed plants, the proposed MS approach consistently outperformed the other databases across all classifiers. The LDA model, in particular, achieved the highest accuracy at 92.3 ± 4.0%, followed by the SVM at 91.6 ± 5.2%, and kNN at 89.1 ± 6.6% on the MS database. These results suggest that the richer spectral information provided by the MS approach significantly enhances the ability of the classifier to distinguish between stressed and non-stressed plants.

The traditional approach based on the computation of vegetation indices also performed well, particularly for the LDA model, which achieved an accuracy of 90.3 ± 4.4%, followed by the SVM at 88.7 ± 4.5%, and kNN at 84.2 ± 3.8%, indicating that the combination of various vegetation indices provides a robust signal for binary stress classification. The LICOR database, which captures physiological measurements, had the lowest overall performance, with kNN and LDA reaching similar levels of accuracy (82.0 ± 6.5% and 82.2 ± 7.5%, respectively), and SVM slightly higher at 83.2 ± 7.4%. This suggests that while physiological data are valuable, the more comprehensive spectral data in the MS and VI databases may capture additional information that aids in stress classification.

The multiclass classification task, which involves distinguishing between different levels of plant stress (NS, STE, MTE, and LTE), presented a more challenging scenario for the classifiers. Nevertheless, the proposed MS approach again led to superior performance across all classifiers. The LDA model performed the best, achieving an accuracy of 70.3 ± 11.2%, followed by the SVM at 70.1 ± 9.4%, and kNN at 60.2 ± 10.3%. These results reinforce the effectiveness of the MS approach in capturing the subtle differences between various stress levels, making it the most suitable database for fine stress classification.

The approach based on the computation of vegetation indices also showed good performance in multiclass classification, particularly with LDA (67.3 ± 10.2%), followed by SVM (60.3 ± 6.8%) and kNN (48.3 ± 5.8%), though slightly lower than the MS database. The LICOR database, however, struggled in this task, with accuracies at most 56.5% across all classifiers. This indicates that while LICOR measurements may be sufficient for binary classification, they may lack the necessary resolution or breadth of information required to differentiate between multiple stress levels.

In terms of feature selection, the number of selected features varied significantly across different databases and models.

For the binary classification, the number of features selected for LDA, SVM, and kNN is generally fewer. For instance, LDA uses only 1 feature for the LICOR method, 27 for Vegetation Indices, and 47 for the proposed MS method.

Conversely, for the multiclass problem, the number of features required increases significantly across all models and methods when compared to binary classification. LDA, for example, requires 2 features for LICOR but 28 for Vegetation Indices and 52 for the proposed MS method. Similar trends are observed with SVM and kNN, where more features are necessary in multiclass cases (SVM requires up to 48 features and kNN up to 47 for the proposed MS method).

The requirement for a higher number of features in multiclass scenarios can be attributed to the additional complexity in distinguishing between multiple classes, which often cannot be captured with the fewer features that might suffice in a binary classification context.

**Figure 6:**
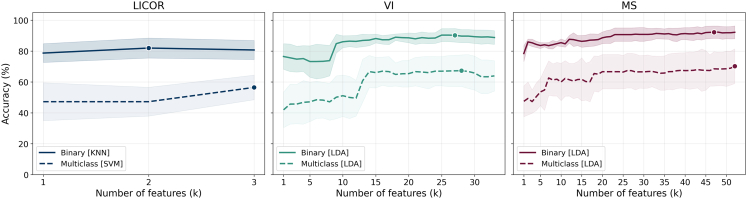
Classification accuracy of the best performing models as a function of the number of selected features for the LICOR, VI, and MS databases

Figure 6 shows how the accuracy of the best performing models varies with the number of selected features for the LICOR, VI, and MS databases, respectively. As emerges from the figure, the LICOR database exhibited minimal improvement as more features were added, indicating limited information for classification. The VI database shows sharp improvement with 8 features for the binary problem and 12 features for the multiclass problem before declining, suggesting diminishing returns and possible overfitting. In contrast, the MS database consistently improved with more features, reaching its highest accuracy at 47 features, before slightly declining for the binary problem only.

**Figure 7:**
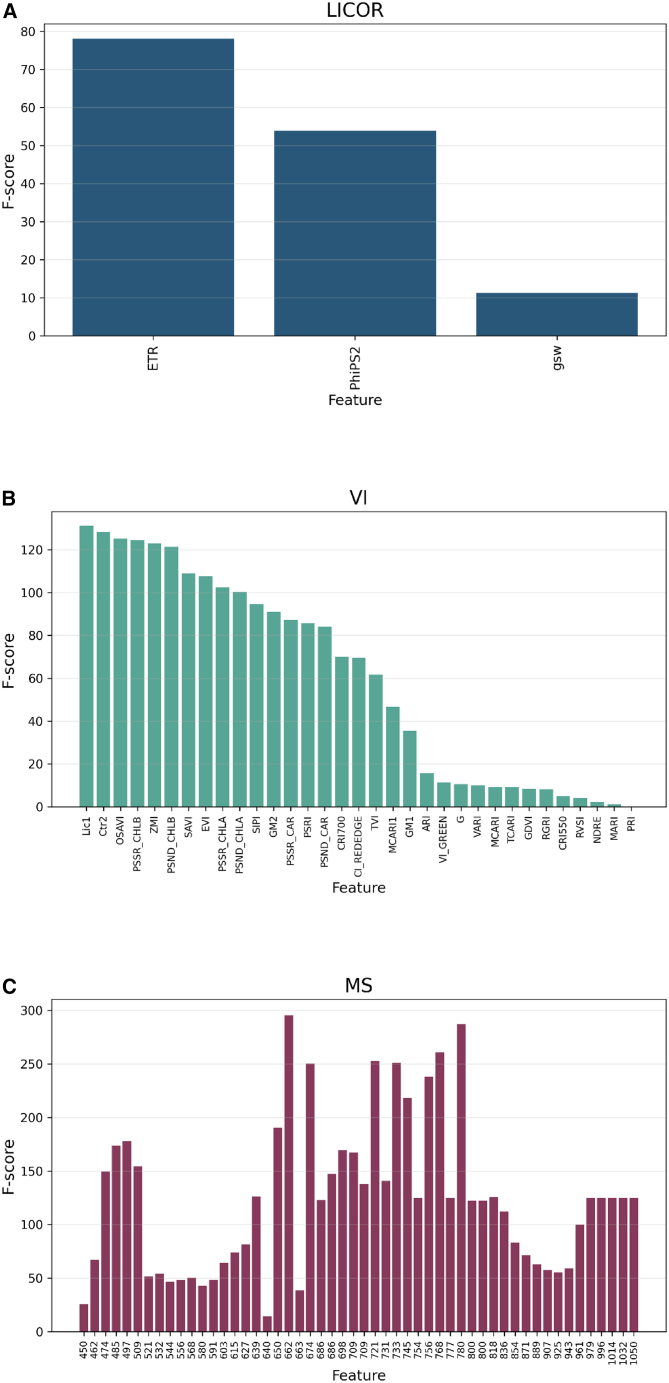
Feature importance computed with the KBest method for each dataset (A) For the LICOR, features are placed in decreasing order of importance; (B) for the VI datasets, features are placed in decreasing order of importance; (C) for the MS dataset, features are placed in order of increasing wavelength.

Figure 7 shows the most informative features for each dataset. For the LICOR dataset, ETR holds most information, followed by Φ_*PSII*_ and gsw, which is the only feature that was not used for binary classification. Among the vegetation indices, Lic1 was the most informative, followed by Ctr2 and OSAVI, while PRI does not hold any information for classification purposes. With the MS method, the most informative wavelengths are 662 nm and 780 nm, with most of the information present in the range between these two wavelengths, implying that the near-infrared range is the most useful for stress identification.

**Figure 8:**
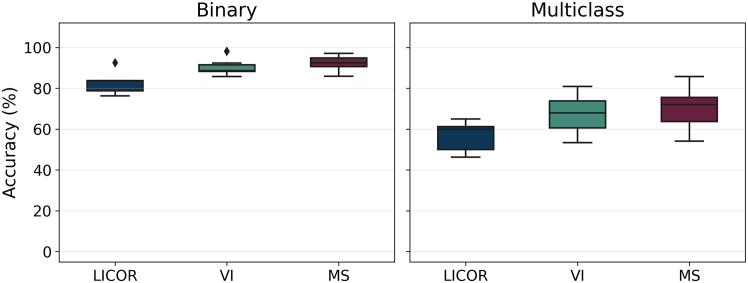
A boxplot of the best-performing models' accuracy achieved for each classification problem (i.e., binary and multiclass) and input database (i.e., LICOR, VI, and MS) For the LICOR dataset, the optimal model is SVM; for the VI and MS datasets, the optimal model is LDA.

**Figure 9:**
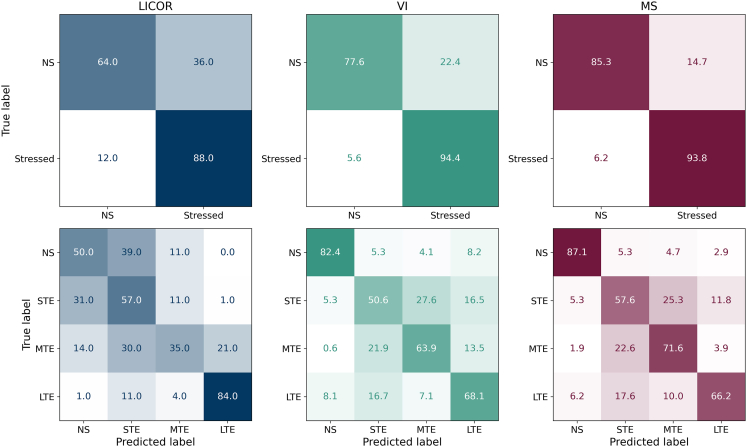
Confusion matrix resulted from the best performing models’ validation for each classification problem (i.e., binary and multiclass) and input database (i.e., LICOR, VI, and MS) For the LICOR dataset, the optimal model is SVM; for the VI and MS datasets, the optimal model is LDA.

Figures 8 and 9 present the classification accuracy and confusion matrix, respectively, achieved by the best-performing model, i.e., the SVM for the LICOR dataset and the LDA for the VI and the MS datasets, across different databases in both binary and multiclass classification tasks. For brevity, the classification accuracy and confusion matrix achieved by the other models are not shown, since they achieved worse performance for all three databases, as emerges from Table 1.

For the binary classification, the MS database outperformed both LICOR and VI databases. The accuracy for the MS database is consistently higher (92.3 ± 4.0%), reflecting the superior ability of LDA when fed with the rich spectral information of the MS database. In comparison, the VI database achieved moderately high accuracy but with more variance (90.3 ± 4.4), while LICOR showed the lowest accuracy (83.2 ± 7.4), indicating its limited capacity for binary plant stress classification.

In the multiclass classification, the MS database again showed the highest accuracy (70.3 ± 11.2%), outperforming both the VI and LICOR databases. The VI database demonstrated moderate performance (67.3 ± 10.2), while the LICOR database exhibited the lowest accuracy (56.5 ± 8.0). These results suggest that the MS database provides more detailed and reliable information for distinguishing between different stress levels in plants, while the VI database offers moderate performance, and LICOR struggles with more complex classification tasks.

## Discussion

In this work, a multispectral device to be used either in hand or integrated into a robotic arm for in-field plant stress detection has been proposed. The device includes a broadband LED, two VIS-NIR multispectral sensors with 52-wavelength-resolution, and advanced methods for plant stress detection, namely SelecKBest for feature selection from raw multispectral signals and kNN for the classification of stress severity based on the computed features.

The performance of the proposed multispectral device was evaluated through experimental validation carried out on tobacco plants under salinity stress and compared to the ones obtained by typical literature approaches, i.e., low-resolution multispectral devices based on the computation of vegetation indices and LICOR porometer/fluorometer.

The results of the classification experiments demonstrated the high accuracy of the proposed multispectral device in comparison to traditional literature approaches. In both binary (“stress” and “no stress”) and multiclass classification tasks (“no stress”, “short-term exposure”, “medium-term exposure” and “long-term exposure”), the proposed approach demonstrated higher accuracy across all the tested models (LDA, SVM and kNN), particularly with the LDA classifier, which achieved a mean accuracy of 92.3 ± 4.0% in the binary task and 70.3 ± 11.2% in the multiclass task.

In contrast, the approach based on the computation of vegetation indices demonstrated reasonable performance with greater variability in the binary task (90.3 ± 4.4%), but less in the multiclass task (67.3 ± 10.2%). This highlights its limitations in comparison to the proposed multispectral device, which uses a broader set of spectral bands. The LICOR device consistently underperformed across both tasks (83.2 ± 7.4% and 56.5 ± 8.0 for binary and multi-class problems, respectively), further demonstrating that physiological measurements alone do not capture the same breadth of information as spectral data. These findings underscore the potential of multispectral data to accurately detect and classify plant stress, offering a more reliable approach for accurate plant stress severity classification.

It is worth pointing out that the comparison between the proposed multispectral (MS) approach and the LI-COR system, which rely on feature sets with different dimensionality, is intended to evaluate two different sensing paradigms rather than enforcing a same-dimension analysis. While LI-COR provides a limited set of physiological indicators, the MS approach exploits a richer spectral representation combined with automatic feature selection, resulting in improved classification performance. Future work will investigate reduced-dimensional comparisons to better assess the role of feature richness versus feature quality.

From a practical perspective, the proposed device is designed to operate in a controlled measurement configuration, ensuring repeatable and reliable data acquisition. The measurement procedure is fast and non-destructive, allowing multiple leaves to be sampled within a short time. Moreover, the integration of the light source and sensors within a compact enclosure minimizes the influence of external environmental conditions, making the system suitable for in-field applications. These characteristics, combined with the low cost and portability of the device, support its potential adoption in precision agriculture scenarios for early stress monitoring.

Future works will be mainly addressed to: 1) investigating longer monitoring periods to assess the applicability of the approach to long-term stress conditions; 2) expanding the database to include a larger variety of plants and stress typology; 3) integrating the proposed device into a robotic arm; 4) improving the user experience through the integration of an intuitive interface, compatible with laptops, iPads, or smartphones, and designed to be user-friendly for individuals with diverse technical skills; and 5) validating the overall platform for plant stress detection in simulated and real-world scenarios.

### Limitations of the study

A limitation of the present study is that the experimental validation was conducted under acute salinity stress conditions over a 6-day period. Therefore, the proposed model has been validated within this temporal window and may not directly generalize to chronic stress scenarios typically encountered in real agricultural settings.

## Resource availability

### Lead contact

Requests for further information and resources should be directed to and will be fulfilled by the lead contact, Clemente Lauretti (c.lauretti@unicampus.it).

### Materials availability

This study did not generate new materials.

### Data and code availability

• Data reported in this paper will be shared by the lead contact upon request.
• Codes reported in this paper will be shared by the lead contact upon request.
• Any additional information required to reanalyze the data reported in this paper is available from the lead contact upon request.

## Acknowledgments

This work was supported by the 10.13039/501100003407Italian Ministry of Universities and Research (Mur) with the project FUTURE AI RESEARCH (FAIR) CUP: C53C22000800006. Sara Cimini, Laura De Gara, and Marco Santonico are part of Agritech National Research Center and received funding from the European Union 10.13039/100031478NextGenerationEU (PIANO NAZIONALE DI RIPRESA E RESILIENZA (PNRR)—MISSIONE 4 COMPONENTE 2, INVESTIMENTO 1.4—D.D. 1032 17/06/2022, CN00000022). This manuscript reflects only the authors’ views and opinions; neither the European Union nor the European Commission can be considered responsible for them.

## Author contributions

Conceptualization, C.L., S.C., A.Z., L.D.G., G.P., M.S., and L.Z.; methodology, C.L., C.T., and A.Z.; investigation, C.L., S.C., B.P., and M.P.; writing – original draft, C.L., S.C., and C.T..; writing – review and editing, A.Z., B.P., M.P., L.D.G., G.P., M.S., and L.Z.; funding acquisition, L.D.G., G.P., M.S., and L.Z.; resources, L.D.G., G.P., M.S., and L.Z.; supervision, L.D.G., G.P., M.S., and L.Z.

## Declaration of interests

The authors declare no competing interests.

## STAR★Methods

### Key resources table

**Table:** 

REAGENT or RESOURCE	SOURCE	IDENTIFIER
**Biological samples**		

Tobacco (Nicotiana tabacum)	N/A	N/A

**Critical commercial assays**		

LI-600 porometer/fluorometer	LI-COR	N/A

**Software and algorithms**		

Python version 3.12	Python Software Foundation	https://www.python.org
MATLAB	MATLAB R2023a	https://it.mathworks.com/
Linear Discriminant Analysis	Zapolska et al.^60^	N/A
Support Vector Machine	Karthickmanoj et al.^61^	N/A
k-Nearest Neighbors	Zahid et al.^62^	N/A
SelectKBest	Luepsen^37^	N/A

**Other**		

SMB1N-BB450 LED	Roithner Lasertechnik GmbH, Austria	N/A
C12880MA	Hamamatsu Photonics K.K., Japan	N/A
C14384MA	Hamamatsu Photonics K.K., Japan	N/A

### Experimental model and study participant details

The study involved 12 Tobacco (*Nicotiana tabacum*) plants sown in soil at 25 ± 1°C with a photoperiod of 16-h light/8-h dark. Stress condition was produced by irrigating with double-distilled water at concentrations of 100 mM of sodium chloride two months after germination for 6 days. Multispectral data and porometer/fluorometer signals from both the control and treated groups of plants were acquired daily, with 12 measurements recorded per plant each day, each on four different leaves per plant. This was done to provide a representative sampling of the plant while maintaining a reasonable acquisition time and ensuring experimental repeatability. Leaves were selected to ensure consistency across plants (e.g., similar developmental stage). For each leaf, measurements were performed in predefined areas, and multiple scans were acquired to reduce local variability and improve robustness. A total of three scans were measured at each position, and the measurements from the three positions were averaged as one sample.

### Method details

#### Hardware components

The proposed multispectral device is composed of i) a SMB1N-BB450 broadband LED for illuminating the target leaf with a broad spectrum of light that spans wavelengths from 340 to 1050 nm, ii) two multispectral sensors (the C12880MA and the C14384MA), iii) a printed circuit board featuring microcontrollers, analog-to-digital converters, and additional circuitry powered at 5V, consuming 235 mW in idle mode and 740 mW during data collection, and iv) a 3D printed enclosure.

#### Software components

The device includes software components designed to identify stress conditions with the collected multispectral data. The signal is first filtered with a moving average filter with a 3-sample buffer and normalized using a white and a dark reference. Then, a feature selection featuring the SelectKBest method is applied to select k features, i.e. wavelengths, based on their statistical significance according to the ANOVA F-value. This ensures the choice of only the most informative features to perform dimensionality reduction and to optimize the balance between the quantity of informative data and model performance. Finally, a supervised machine-learning model is intended to detect plant stress and classify its severity. To determine the optimal pipeline configuration, three different state-of-the-art machine-learning approaches have been compared. They are: i) the Linear Discriminant Analysis (LDA), ii) the Support Vector Machine (SVM), and iii) the k-Nearest Neighbors (kNN).

### Quantification and statistical analysis

The experimental dataset consisted of measurements acquired from n = 12 plants (6 control and 6 treated). For each plant, multispectral and porometer/fluorometer measurements were collected (12 measurements per day over 6 days, on four leaves per plant), and repeated scans were averaged to obtain a single sample, as detailed in the Experimental Validation section.

All data were grouped into four stress classes: no stress (NS), short-term exposure (STE), medium-term exposure (MTE), and long-term exposure (LTE), as defined in the data acquisition and database building procedure (Figure 3). For each class, mean value and standard deviation (SD) were computed. A visual representation of multispectral data and porometer/fluorometer measurements is reported in Figures 4 and 5, respectively. To assess statistically significant differences between groups, a Mann-Whitney U test was applied due to the non-normal distribution of the data, followed by Bonferroni correction for multiple comparisons as reported in the Experimental Protocol section. Statistical significance of the comparison among the different groups is reported in Figure 5 through asterisks (∗ p ≤ 0.05, ∗∗ p ≤ 0.01, ∗∗∗ p ≤ 0.001, and ∗∗∗∗ p ≤ 0.0001).

Feature selection was performed using a univariate ANOVA F-test. Feature ranking was computed once on the entire dataset before cross-validation, producing a global ordered list of features based on their discriminative power. An incremental feature selection strategy was then applied: starting from the most informative feature, additional features were progressively included according to the ranking, without reordering previously selected ones. This allowed the systematical analisis of the impact of the number of features on model performance and to identify the optimal feature subset. The optimal number of selected features obtained through SelectKBest (ANOVA F-test), is reported in Table 1 and further analyzed in Figures 6 and 7.

Two classification problems were evaluated: (i) binary classification (non-stressed vs stressed plants), and (ii) multiclass classification (NS, STE, MTE, LTE). Model performance was assessed using a leave-one-group-out (LOGO) cross-validation strategy, where each group corresponds to a single plant, ensuring independence between training and test sets. For each fold, the model was trained on *n* − 1 plants and tested on the held-out plant, as described in the Classification model training and testing section. Performance was evaluated using accuracy (%), reported as mean ± standard deviation across folds, see obtained results in Table 1 and Figure 8.

## Figures and Tables

**Table 2:** Vegetation indices for plant phenotyping that were computed through the proposed multispectral device

Index Name	Description	Equation	Reference
NDVI	normalized difference vegetation index	$\frac{r_{800}-r_{670}}{r_{800}+r_{670}}$	Tian et al.^29^
MCARI1	modified chlorophyll absorption in reflectance index 1	1.2 × (2.5 × (*r*_790_ − *r*_670_) − 1.3 × (*r*_790_ − *r*_550_))	Haboudane et al.^39^
OSAVI	optimized soil-adjusted vegetation index	$\frac{\left(1+0.16\right)\times \left(r_{790}-r_{670}\right)}{r_{790}+r_{670}+0.16}$	Rondeaux et al.^40^
G	greenness index	$\frac{r_{554}}{r_{677}}$	Modarelli et al.^41^
MCARI	modified chlorophyll absorption in reflectance index	$\left(\frac{r_{700}-r_{670}}{r_{700}}\right)-0.2\times \left(\frac{r_{700}-r_{550}}{r_{700}}\right)$	Daughtry et al.^42^
TCARI	transformed chlorophyll absorption in reflectance index	$3\times \left(\frac{r_{700}-r_{670}}{r_{700}}\right)-0.2\times \left(\frac{r_{700}-r_{550}}{r_{700}}\right)$	Haboudane et al.^43^
TVI	triangular vegetation index	0.5 × (120 × (*r*_750_ − *r*_550_) − 200 × (*r*_670_ − *r*_550_))	Broge et al.^44^
ZMI	zarco-tejada & miller index	$\frac{r_{750}}{r_{710}}$	Zarco-Tejada et al.^45^
PRI	photochemical reflectance index	$\frac{r_{531}-r_{570}}{r_{531}+r_{570}}$	Gamon et al.^46^
Ctr2	carter index 2	$\frac{r_{695}}{r_{760}}$	Carter et al.^47^
Lic1	lichtenthaler index 1	$\frac{r_{790}-r_{680}}{r_{790}+r_{680}}$	Lichtenthaler et al.^48^
SIPI	structure-independent pigment index	$\frac{r_{800}-r_{450}}{r_{800}+r_{650}}$	Penuelas et al.^33^
GM1	gitelson and merzlyak index 1	$\frac{r_{750}}{r_{550}}$	Gitelson et al.^38^
GM2	gitelson and merzlyak index 2	$\frac{r_{750}}{r_{700}}$	Gitelson et al.^38^
ARI	anthocyanin reflectance index	$\left(\frac{1}{r_{550}}\right)-\left(\frac{1}{r_{700}}\right)$	Gitelson et al.^49^
CI_REDEDGE	chlorophyll index rededge	$\frac{r_{800}}{r_{700}}-1$	Gitelson et al.^50^
CRI550	carotenoid reflectance index 550	$\left(\frac{1}{r_{510}}\right)-\left(\frac{1}{r_{550}}\right)$	Gitelson et al.^49^
CRI700	carotenoid reflectance index 700	$\left(\frac{1}{r_{510}}\right)-\left(\frac{1}{r_{700}}\right)$	Gitelson et al.^49^
EVI	enhanced vegetation index	$\frac{2.5\times \left(r_{800}-r_{670}\right)}{r_{800}+6\times r_{670}-7.5\times r_{480}+1}$	Huete et al.^51^
GDVI	green difference vegetation index	$\frac{r_{800}-r_{550}}{r_{800}+r_{550}}$	Agapiou et al.^31^
MARI	modified anthocyanin reflectance index	$\left(\frac{1}{r_{550}}-\frac{1}{r_{700}}\right)\times r_{800}$	Gitelson et al.^52^
NDRE	normalized difference red edge index	$\frac{r_{790}-r_{720}}{r_{790}+r_{720}}$	Barnes et al.^53^
PSND_CHLA	pigment specific normalized difference for chlorophyll *a*	$\frac{r_{800}-r_{680}}{r_{800}+r_{680}}$	Blackburn et al.^54^
PSND_CHLB	pigment specific normalized difference for chlorophyll *b*	$\frac{r_{800}-r_{635}}{r_{800}+r_{635}}$	Blackburn et al.^54^
PSND_CAR	pigment specific normalized difference for carotenoids	$\frac{r_{800}-r_{470}}{r_{800}+r_{470}}$	Blackburn et al.^54^
PSRI	plant senescence reflectance index	$\frac{r_{678}-r_{500}}{r_{750}}$	Merzlyak et al.^55^
PSSR_CHLA	pigment specific simple ratio for chlorophyll *a*	$\frac{r_{800}}{r_{680}}$	Blackburn et al.^54^
PSSR_CHLB	pigment specific simple ratio for chlorophyll *b*	$\frac{r_{800}}{r_{635}}$	Blackburn at al.^54^
PSSR_CAR	pigment specific simple ratio for carotenoids	$\frac{r_{800}}{r_{470}}$	Blackburn et al.^54^
RGRI	red:green ratio index	$\frac{r_{670}}{r_{560}}$	Gamon et al.^56^
RVSI	red-edge vegetation stress index	$\frac{\left(r_{714}+r_{752}\right)}{2}-r_{733}$	Merton et al.^57^
SAVI	Soil-adjusted vegetation index	$\frac{1.5\times \left(r_{800}-r_{680}\right)}{r_{800}+r_{680}+0.5}$	Huete et al.^58^
VARI	visible atmospherically resistant index	$\frac{r_{550}-r_{670}}{r_{550}+r_{670}-r_{480}}$	Gitelson et al.^59^
VI_GREEN	vegetation index green	$\frac{r_{550}-r_{670}}{r_{550}+r_{670}}$	Gitelson eta l.^59^

## Appendix

### Vegetation indices

Vegetation indices are mathematical formulas that combine reflectance values at specific wavelengths to assess various characteristics of plant health. These reflectance values are denoted as *r*_*λ*_, where *λ* represents the wavelength in nanometers. Table 2 reports a list of vegetation indices for plant phenotyping that could be computed with the proposed multispectral device.

### Machine learning models

In this work, three machine learning approaches were used for binary and multiclass classification. In the following, each approach is detailed.

• Linear Discriminant Analysis (LDA): LDA is a linear classifier that assumes that different classes generate data based on Gaussian distributions with the same covariance matrix. It seeks to find the linear combination of features that best separates the classes by maximizing the ratio of between-class variance to within-class variance. LDA is particularly effective when class distributions are roughly Gaussian and linearly separable. However, its performance degrades when the classes are not linearly separable or when the assumption of equal covariance matrices across classes is violated.^60^
• Support Vector Machine (SVM): SVM is a robust and versatile classifier that constructs an optimal hyperplane to separate classes by maximizing the margin between the nearest data points of different classes (support vectors). The regularization parameter *C* controls the trade-off between maximizing the margin and allowing classification errors, providing flexibility in handling noise and outliers. SVM is particularly powerful for high-dimensional spaces and is effective in cases where the data is not linearly separable, thanks to the kernel trick. However, SVM can be computationally expensive, especially for large databases, and its performance heavily depends on the choice of kernel and tuning of hyperparameters.^61^
• k-Nearest Neighbors (kNN): kNN is a non-parametric, instance-based learning algorithm that classifies a data point by a majority vote among its *k* nearest neighbors in the feature space. It is simple and intuitive, requiring no explicit training phase. kNN works well in cases where decision boundaries are irregular or non-linear. However, kNN is sensitive to noisy data and irrelevant features, making feature selection or dimensionality reduction crucial for its success.^62^
